# Deciphering Clinicoradiologic Phenotype for Thymidylate Synthase Expression Status in Patients with Advanced Lung Adenocarcinoma Using a Radiomics Approach

**DOI:** 10.1038/s41598-018-27273-9

**Published:** 2018-06-12

**Authors:** So Won Lee, Hyunjin Park, Ho Yun Lee, Insuk Sohn, Seung-Hak Lee, Jun Kang, Jong-Mu Sun, Myung-Ju Ahn

**Affiliations:** 1 Department of Radiology and Center for Imaging Science, Samsung Medical Center, Sungkyunkwan University School of Medicine, Seoul, Korea; 2 Department of Radiology, CHA Gangnam Medical Center, CHA University School of Medicine, Seoul, Korea; 3 School of Electronic and Electrical Engineering, Sungkyunkwan University, Suwon, Korea; 4 Center for Neuroscience Imaging Research, Institute for Basic Science, Suwon, Korea; 5 Statistics and Data Center, Samsung Medical Center, Seoul, Korea; 6 Department of Electronic Electrical and Computer Engineering, Sungkyunkwan University, Suwon, Korea; 7 Department of Pathology, Inchun St. Mary’s Hospital, College of Medicine, Catholic University of Korea, Inchun, Korea; 8 Division of Hematology-Oncology, Department of Internal Medicine, Samsung Medical Center, Sungkyunkwan University School of Medicine, Seoul, Korea

## Abstract

We aimed to identify predictive clinicoradiologic characteristics of thymidylate synthase (TS) expression status in advanced non-squamous non-small cell lung cancer patients. We reviewed clinicoradiologic features of 169 patients stratified into TS-negative (n = 84) and TS-positive (n = 85) groups, including quantitative CT radiomic features of both primary lung and metastatic lesions from initial CT and PET. Clinical factors including age and smoking history were significantly associated with TS as well as radiomic features. The predictive performance for dichotomizing TS expression status was slightly higher when imaging features of primary lung lesions were added compared to the model based solely on the clinical features, but without statistical significance (10-fold cross-validated AUC = 0.619 and 0.581, respectively; *P* = 0.425). The predictive performance of clinicoradiologic parameters slightly increased with primary lung lesions only compared to the inclusion of metastatic lesions, but without statistical significance (10-fold cross-validated AUC = 0.619 and 0.554, respectively; *P* = 0.203). Overall survival was prolonged in the TS-negative group compared to the TS-positive group (*P* = 0.001). TS-negativity is a potential prognostic biomarker, and our study presents that although CT radiomic features have potential for predicting TS expression status, clinical significance is uncertain. The addition of radiomic features to clinical factors did not show significant improvement in predicting TS-negativity.

## Introduction

Lung cancer is one of the leading causes of cancer-related mortality in many countries, and non-small cell lung cancer (NSCLC) accounts for approximately 80% of all lung cancers^1^. For advanced NSCLC, platinum-based doublet chemotherapy remains the standard first-line chemotherapy, especially in tumors not harboring either epidermal growth factor receptor (EGFR) mutation or anaplastic lymphoma kinase (ALK) translocation^2^. However, given that patients with metastatic disease have a 5-year survival rate less than 4% in the US^3^, the treatment efficacy and survival outcome of platinum-based doublet chemotherapy are still limited^4^. Therefore, identifying a biomarker that may be helpful in determining a treatment regimen or predicting prognosis in NSCLC patients, ultimately for improvement in survival rate, is needed.

Thymidylate synthase (TS) is a key enzyme in Deoxyribonucleic acid (DNA) synthesis and is also the main target of antifolate drugs including pemetrexed. Pemetrexed, which is increasing its therapeutic scope from second-line therapy to first-line and maintenance therapy, is a multi-targeted antifolate drug that inhibits at least three enzymes including TS involved in DNA synthesis and folate metabolism, resulting in antitumor effects^5,6^.

Previous studies have reported that lower TS expression level is associated with better clinical outcomes for pemetrexed-based chemotherapy in NSCLC^7–10^. A recent phase III study showed survival differences between histologic types; pemetrexed/cisplatin was superior for survival compared with gemcitabine/cisplatin in nonsquamous NSCLC, although it was inferior in squamous cell histology^11^. This result can also be explained by the higher TS expression level in squamous cell carcinoma compared with other histologic types including adenocarcinoma^12^. Moreover, several studies have shown the association between low TS expression level and prolonged overall survival in NSCLC regardless of treatment regimen. Therefore, TS expression itself may be a potential prognostic factor^13–15^.

Regarding the evaluation of TS expression, different methods have been used including immunohistochemistry (IHC) to detect TS expression at the protein level and real-time reverse transcriptase polymerase chain reaction (RT-PCR) at the messenger ribonucleic acid (mRNA) level. Although IHC has been widely used and has shown better correlation with objective response rate prediction in patients with lung cancer receiving pemetrexed-based treatments, factors like tumor heterogeneity, subjective scoring system, or cutoff value are considered limitations, for which cautious data interpretation is required^16^. Also, the assessment of TS expression requires a tumor biopsy, which may not be possible for every primary tumor site. Therefore, a noninvasive imaging biomarker aiding in the prediction of TS expression level would be of great clinical importance.

Thus, we aimed to evaluate the clinicoradiologic features of NSCLC stratified by TS expression status and to correlate those with the prognosis in order to identify useful predictive imaging characteristics of TS expression status and to help develop improved treatment strategies.

## Materials and Methods

Our institutional review board approved this retrospective study with a waiver of informed consent (SMC #2015-07-149). This study was performed in accordance with the principles of the Declaration of Helsinki for medical research involving human subjects.

### Patients

We identified 315 patients who had been histologically diagnosed with advanced non-squamous NSCLC (stage IIIB, IV, or recurrent disease after complete resection) at Samsung Medical Center (Seoul, Korea) from July 2011 to January 2014, who were originally included in a biomarker-stratified randomized phase II trial^17^. All patients had immunohistochemical analysis results for TS expression of biopsy specimens and chest computed tomography (CT) within 3 weeks. Twenty-six patients who had no follow-up CT to evaluate the prognosis were excluded. Twenty-nine patients were excluded for CT review-related factors: non-contrast CT only, images that could not be used for quantitative CT analysis due to the raw dicom data error, or no measurable lesion on CT by RECIST 1.1^18^. Considering the availability of ^18^F-fluoro-2-deoxyglucose (FDG)- positron emission tomography (PET) imaging, 169 of 260 patients were included in the present analysis. Four of 260 patients who had histology other than adenocarcinoma; pleomorphic carcinoma or not otherwise specified, were excluded due to prognosis or image-related factors described above. Therefore, the histology of all 169 patients included was adenocarcinoma.

All patients were stratified into TS-negative or TS-positive groups. Patients for each group were randomly assigned to receive platinum doublet chemotherapy with or without pemetrexed: either intravenous pemetrexed (500 mg/m^2^; Alimta, Eli Lilly, Indianapolis, IN, USA) plus cisplatin (70 mg/m^2^; Cisplan, Dong-A ST, Seoul, Korea) intravenously on day 1 or gemcitabine (1000 mg/m^2^; Gemzar, Eli Lilly, Indianapolis, IN, USA) on days 1 and 8 plus cisplatin (70 mg/m^2^) intravenously on day 1. The administration was repeated every 3 weeks, and patients received a maximum of 6 cycles of chemotherapy until disease progression, unacceptable adverse event, or decision by the patient or physician. The response was assessed by CT scans every two cycles according to RECIST 1.1^18^.

### Imaging and Analysis

Imaging characteristics of each lesion were evaluated using chest CT and the PET component of PET/CT. Dedicated chest CT images were obtained with several multi-detector CTs including 8-, 16-, or 64-detector row CT scanners. All CT exams were performed with contrast. Chest CT data were interfaced directly to a picture archiving and communication system (PACS) (Path-Speed or Centricity 2.0; GE Healthcare, Mt. Prospect, IL, USA), which displayed all image data on two monitors (1536 × 2048 matrix, 8-bit viewable grayscale, 60-foot-lambert luminescence). The monitors were adapted to view both mediastinal (width, 400 HU; level, 20 HU) and lung (width, 1500 HU; level, −700 HU) window images.

Three radiologists (H.Y.L., H.S.H., and E.Y.K.) blinded to the information about the TS group and treatment allocation performed independent reviews of the radiological data from all randomized patients with at least a baseline and one follow-up scan and evaluated treatment response prospectively in consensus. A maximum of five lesions including primary lung lesion for each patient were included and measured at baseline CT according to RECIST guidelines^18^.

A thoracic radiologist (S.W.L, with six years of experience in thoracic CT interpretation) who was unaware of the clinical data and histologic diagnoses retrospectively evaluated the initial CT scans. The size and opacity of the primary lung lesion, the presence/absence of pleural metastases and lymphangitic carcinomatosis, and the presence and location of distant metastases were evaluated as CT features. The opacity of primary lung lesion was characterized as either solid (lesions with increased opacity obscuring pulmonary vessels), non-solid (lesions with ground glass opacity preserving the bronchial and vascular margins), or part-solid (lesions with mixed solid and ground glass opacity components).

For quantitative CT analyses, tumors were segmented by drawing a region of interest (ROI) covering the largest possible area of the whole lesion (Figure S1). Not only the primary lung lesions, but also the metastatic lesions were segmented by drawing a ROI. We used Mricro software (http://www.cabi.gatech.edu/mricro/mricro/ version 1.40) to draw the ROIs. Next, voxel-based CT numbers were collected from lesion segmentations.

**Figure 1:**
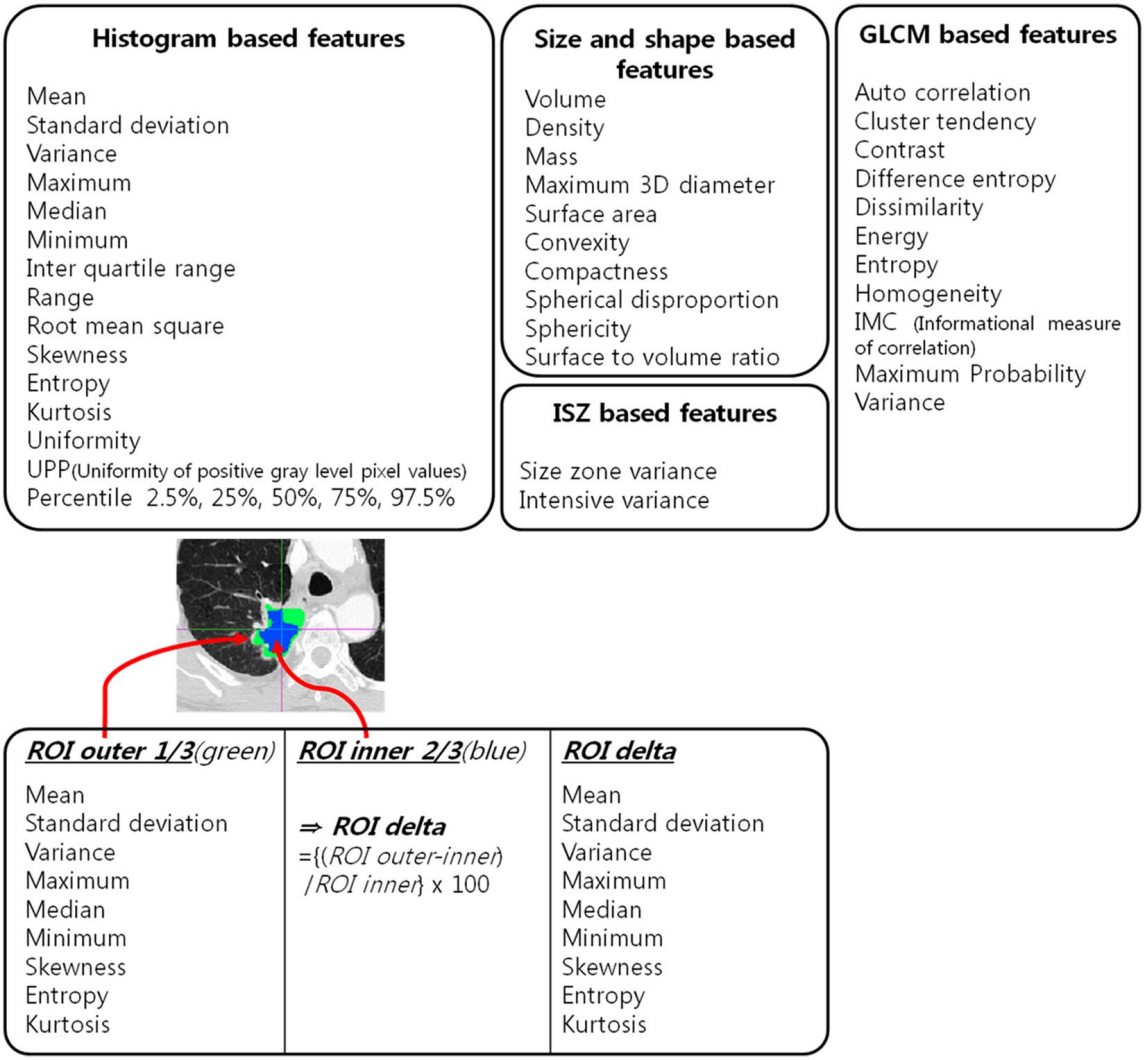
A total of 60 CT radiomic features used in this study. The features were classified into four categories of histogram-based, shape-based, gray level co-occurrence matrix (GLCM), and intensity size zone (ISZ) features.

We computed radiomic features from raw imaging data over an ROI drawn by a thoracic radiologist (S.W.L, with six years of experience in thoracic CT interpretation)^19^. A total of 60 features were computed in our study(Fig. 1). The features were classified into four categories: histogram-based, shape-based, gray level co-occurrence matrix (GLCM), and intensity size zone (ISZ) features. Histogram-based features were computed using the intensity (HU) distribution of a given ROI. These features reflect intensity information of a given ROI. For histogram features, we adopted 2 types of histograms as follows: (1) regular histogram of all intensity values within the ROI (19 features); (2) histogram based on inner and outer portions of the ROI (18 features). A given ROI was partitioned into inner ROI (2/3 of the whole ROI volume) and outer ROI (1/3 of the whole ROI volume) purely based on the volume. We hypothesized that partitioning the outer part of the ROI would better reflect the aggressiveness of the tumor than the total ROI, which may have an offset portion as a whole. As the volume of the tumor decreased with this partitioning procedure, some of the features were not analyzable separately for inner or outer portion of the tumor due to its small volume. Therefore, inner and outer ROIs each had nine features. The difference between inner and outer ROI value was defined as delta ROI, which may signify intra-tumor heterogeneity. A total of 9 features were included for both outer ROI and delta ROI. The total number of histogram-based features, including median, energy, min, max, and range, was 37^20^. Shape-based features reflect morphological data of the ROI. A total of 10 features including volume, surface area, convexity, and compactness were computed^19,21^. GLCM-related features consider intensity values of a neighborhood instead of one voxel. One could quantify how similar or dissimilar voxel intensities are within a neighborhood. Thus, GLCM-related features could be used to quantify texture data^22^. Image intensities were discretized to 256 level for robust computation of GLCM, which was consistent with the Freedman-Diaconis formula and other studies^23,24^. GLCMs were computed for 13 directions and the average of 13 matrices were used for feature computation. The GLCM had 11 features including auto-correlation, and the dissimilarity was computed^19^. ISZ features were also correlated to texture data, but could be quantified beyond the immediate neighborhood. They assumed that an ROI could be further divided into sub-regions with uniform intensity but variable size. ISZ could quantify how many sub-regions and how often certain sub-regions occur within the tumor^25,26^. Image intensities were discretized to 32 level for robust computation of ISZ matrix. We chose 32 bins as it provided sensitive matrix to quantify number of sub-regions^26^. A total of two features, intensity variability and size-zone variability, were computed. Details regarding mainly adopted features are provided in the Supplementary Table. To increase the reproducibility of results, most of our radiomic features were computed using open-source platform called *PyRadiomics*^27^, available at www.radiomics.io. Only for 11 features including percentile (2.5%, 25%, 50%, 75% and 97.5%), uniformity of positive gray level pixel values (UPP), density, mass, convexity, size-zone variance and intensive variance, we used in-house MATLAB code (Mathworks Inc., MA, USA). The source codes used for the analysis are provided in the supplementary data. PET/CT was available in 169 patients. Details of PET/CT acquisition are described in Appendix S1.

For semi-quantitative analysis of FDG uptake from PET/CT, ROIs were placed over the most intense area of FDG accumulation. When nodular FDG uptake could not be assessed on PET component images of PET/CT, an ROI was drawn in a presumed nodular location based on CT component images of PET/CT. FDG uptake within the ROIs was calculated as the maximum standardized uptake value (SUVmax).

### Thymidylate Synthase Expression Analyses

All specimens obtained from patients with advanced non-squamous NSCLC were analyzed for TS protein expression by IHC as described previously^10^. The specimens used in this study were 4-µm-thick sections of paraffin-embedded tissue, and monoclonal anti-TS (4H4B1, Invitrogen, Carlsbad, CA) was used for the staining of TS protein. IHC staining of TS was assessed by two investigators (S.Y.H. and J.H.). Based on a previous study using a modified H-score system by Sun *et al*.^10^, we stratified TS-positive or TS-negative tumors by the cutoff value of 10%. TS-positivity was defined as TS expression in more than 10% of tumor cells, and TS-negativity as TS expression in 10% or fewer tumor cells.

### Statistical Analysis

To evaluate the association between clinicoradiologic characteristics and TS expression status from primary lung lesions and mean value of both primary and metastatic lesions, logistic regression analysis was performed. Characteristics with *P*-value less than 0.05 were considered to be statistically significant.

For the analysis of progression-free survival (PFS) and overall survival (OS) according to TS expression status, we used log-rank test with Kaplan-Meier curves. Statistical analyses were performed using SPSS software (SPSS for Windows, version 11.0, 2001; SPSS, Chicago, IL, USA) or MedCalc software (MedCalc for Windows, version 16.1, 2016; MedCalc Software, Ostend, Belgium)

### Internal validation

To evaluate the predictive performance of the prediction model, 10-fold cross-validation (CV) procedure was used as follows:

Step 1. The total data were randomly divided into ten equally sized subsets.

Step 2. A single subset was used as the validation data, and the remaining nine subsets were used as training data.

Step 3. The variables were selected using variable selection procedure based on random forests proposed by Genuer *et al*. in the training set^28^.

Step 4. Random forest was applied to the selected variables to fit a prediction model.

Step 5. A fitted prediction model was applied to the validation data and the predicted probabilities were calculated.

Step 6. Steps 2–5 were repeated ten times.

Step 7. After the cross-validation is complete, the predicted probability values of all patients calculated by 10 fold CV were combined together. A single ROC curve was drawn as in Simon *et al*. and the area under the curve (AUC) value was calculated^29^.

To compare 10-fold cross-validated AUC value of prediction model, the nonparametric approach of DeLong *et al*. was used^30^.

### Data Availability

The datasets analyzed during the current study are available from the corresponding author on reasonable request.

## Results

### Baseline Characteristics

Eighty-one women (47.9%) and 88 (52.1%) men (median age, 59 years) were enrolled. All patients are from Korean population. A total of 296 measurable lesions from 169 patients were analyzed. Among 296 lesions, 169 were primary lung lesions, 93 were metastatic lymph nodes, 15 were pulmonary metastases, 2 were metastatic pleural lesions, and 17 were distant metastases.

On the basis of TS expression status, the TS-negative group consisted of 84 patients, and the TS-positive group included 85 patients (Table 1).

**Table 1:** Patient Characteristics (n = 169).

Characteristic	TS-Negative Group (n = 84)	TS-Positive Group (n = 85)
	No. of Patients (%)	No. of Patients (%)
Age (years)*	58.5 (52.0, 64.8)	60.0 (56.0, 68.0)
Sex		
Female	47 (56.0)	34 (40.0)
Male	37 (44.0)	51 (60.0)
Smoking		
Never smoker	52 (62.0)	35 (41.0)
Ever smoker	32 (38.0)	50 (59.0)
Histologic type		
Adenocarcinoma	84 (100.0)	85 (100.0)
Tumor stage		
IV	84 (100.0)	80 (94.0)
IIIB	0 (0.0)	4 (5.0)
Recurrent	0 (0.0)	1 (1.0)
Chemotherapy		
Pemetrexed/Cisplatin	47 (56.0)	50 (59.0)
Gemcitabine/Cisplatin	37 (44.0)	35 (41.0)
EGFR mutation		
Positive	38 (45.2)	23 (27.1)
Negative	39 (46.4)	59 (69.4)
Unknown	7 (8.3)	3 (3.5)
Follow-up period (months)*	15.0 (14.8, 18.5)	11.4 (12.4, 16.0)

^*^Data are median (interquartile range).

### Analysis Dealing With Only Primary Lung Lesions

The association between imaging parameters and TS expression status was evaluated only for primary lung lesions. Considering the availability of PET/CT imaging, a total of 169 patients with primary lung lesions were included. Younger age and never-smoker status were significantly associated with TS negativity (*P* = 0.003 and *P* = 0.024, respectively). Higher skewness (*P* = 0.041) and lower kurtosis (*P* = 0.005) of the whole tumor and higher skewness_outer_ (*P* = 0.020) and lower kurtosis_outer_ (*P* = 0.016) of histogram-based features were also associated with TS negativity. The root mean square (RMS), mean_outer_, median_outer_, and maximum_delta_ values from histogram-based features and dissimilarity and entropy of GLCM-based features were significantly associated with TS expression status (Table 2). CT features including size (*P* = 0.439) and opacity (*P* = 0.152) of the primary lung lesion, the presence/absence of pleural metastases (*P* = 0.489) and lymphangitic carcinomatosis (*P* = 0.970), and the presence of distant metastases (*P* = 0.785) were not significantly associated with TS expression status. SUVmax from PET/CT was not significantly correlated with TS expression status (*P* = 0.111).

**Table 2:** The result of association between clinicoradiologic variables and TS expression status from primary lung lesions.

Characteristics	TS-Negative Group	TS-Positive Group	OR (95% CI)	P value
Age (yrs)	58.5 (52.0, 64.8)	60.0 (56.0, 68.0)	0.508 (0.280–0.919)	0.003
Smoking history* (%) Never Current or Ex	52 (61.9) 32 (38.1)	35 (41.2) 50 (58.8)	0.209 (0.077–0.566)	0.024
RMS	92.6 (79.6, 129.1)	90.6 (77.8, 107.6)	1.970 (1.089–3.567)	0.008
Skewness	−2.6 (−3.4, −1.6)	−2.7 (−4.0, −1.8)	1.004 (1.000–1.312)	0.041
Kurtosis	15.7 (10.1, 22.4)	20.4 (10.5, 32.5)	0.746 (0.611–0.918)	0.005
Mean_outer_	28.9 (−33.3, 50.3)	36.4 (−6.1, 53.0)	0.976 (0.959–0.992)	0.038
Median_outer_	44.0 (22.0, 58.0)	52.0 (30.0, 62.0)	0.977 (0.959–0.994)	0.020
Skewness_outer_	−2.1 (−2.9, −1.5)	−2.6 (−3.5, −1.7)	1.336 (1.089–1.638)	0.020
Kurtosis_outer_	10.8 (7.1, 19.6)	12.7 (7.3, 25.3)	0.155 (0.044–0.545)	0.016
Maximum_delta_	5.7 (−4.9, 15.2)	12.4 (−0.6, 30.6)	0.604 (0.411–0.889)	0.009
Dissimilarity	10.3 (8.3, 12.5)	9.5 (7.2, 12.9)	1.136 (1.089–1.638)	0.007
Entropy GLCM	10.7 (9.9, 11.1)	10.5 (9.9, 11.0)	1.025 (1.008–1.042)	0.028

Abbreviations: RMS, root mean square; GLCM, gray level co-occurrence matrix.

Unless otherwise indicated, data are median (interquartile range).

^*^Data indicate the number of individuals (percentage).

### Analysis of Combined Data of Primary Lung Lesions and Metastatic Lesions

The association between the imaging parameters and TS expression status was evaluated for the primary lung lesions as well as the metastatic lesions, for which the mean value of primary and metastatic lesions from each patient was used for analysis. Eighty-four patients had more than one metastatic lesion other than the primary lung lesion. The mean number of total lesions in each of all 169 patients was 1.75. Younger age and never-smoker were significantly associated with TS negativity (*P* = 0.043 and *P* = 0.005, respectively). A lower CT attenuation value at the 25^th^ percentile and higher CT attenuation value at the 97.5^th^ percentile from histogram-based features were significantly associated with TS negativity (both *P* < 0.001). Inter-quartile range (IQR), RMS, mean_outer_, median_outer_, and maximum_outer_ values from histogram-based features were also associated with TS expression status (Table 3). SUVmax from PET/CT was not significantly associated with TS expression status (*P* = 0.265).

**Table 3:** The result of association between clinicoradiologic variables and TS expression status from the mean value of both primary and metastatic lesions.

Characteristics	TS-Negative Group	TS-Positive Group	OR (95% CI)	P value
Age (yrs)	58.5 (52.0, 64.8)	60.0 (56.0, 68.0)	0.720 (0.014–0.902)	0.043
Smoking history* Never Current or Ex	52 (61.9) 32 (38.1)	35 (41.2) 50 (58.8)	0.099 (0.022–0.441)	0.005
IQR	67.3 (55.3, 81.9)	60.5 (48.0, 71.5)	1.970 (1.089–3.569)	0.003
RMS	92.0 (79.6, 126.7)	88.4 (76.4, 105.0)	1.591 (1.119–2.779)	0.009
25^th^ percentile (HU)	16.8 (0.3, 32.0)	21.0 (8.1, 36.0)	0.749 (0.611–0.918)	<0.001
97.5^th^ percentile (HU)	137.5 (113.4, 163.0)	135.0 (118.6, 155.0)	1.008 (1.000–1.018)	<0.001
Mean_outer_	29.2 (−8.0, 50.4)	36.4 (1.8, 53.2)	0.959 (0.928–0.991)	0.001
Maximum_outer_	249.9 (201.9, 297.3)	230.0 (184.3, 281.0)	1.877 (1.022–3.448)	0.016
Median_outer_	44.8 (31.3, 59.1)	49.0 (31.8, 63.2)	0.155 (0.044–0.545)	0.003

Abbreviations: IQR, interquartile range; RMS, root mean square.

Unless otherwise indicated, data are median (interquartile range).

^*^Data indicate the number of individuals (percentage).

### Predictive Performance of Clinical and Imaging Parameters for Dichotomizing TS Expression Status

To compare the predictive performance of clinical and imaging parameters for dichotomizing TS expression status, 10- fold cross-validation (CV) procedure was used.

**Figure 2:**
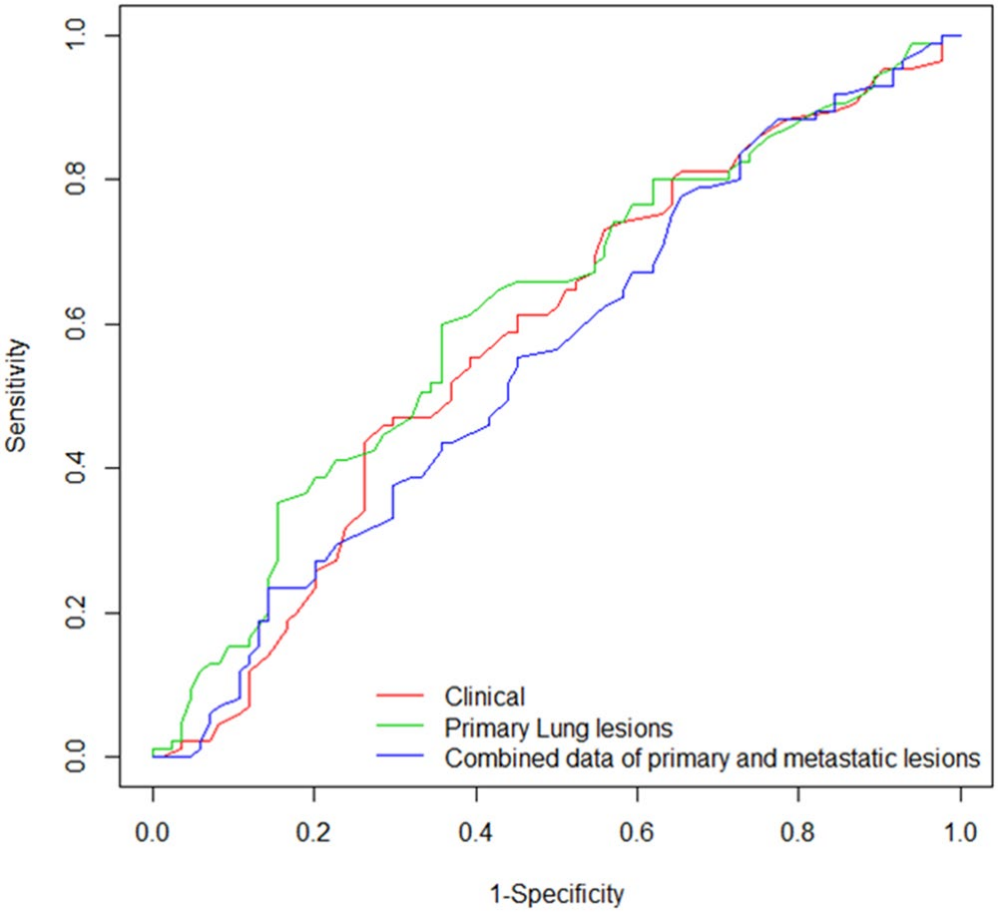
Comparison of ROC curves for dichotomizing TS expression status between the model using clinical features and the model with addition of radiomic features. The red line represents the model using clinical variables only (10-fold cross-validated AUC = 0.581). The green line represents the model using both clinical variables and radiomic features from primary lung lesions (10-fold cross-validated AUC = 0.619). The blue line represents the model using both clinical variables and radiomic features from the combined data of primary and metastatic lesions (10-fold cross-validated AUC = 0.554).

10-fold cross-validated AUC of the prediction models using only clinical variables, both clinical variables and radiomic features from primary lung lesions, and both clinical variables and radiomic features from the combined data of primary and metastatic lesions were 0.581, 0.619, and 0.554, respectively (Fig. 2).

The performance of predicting the TS expression status was slightly higher when radiomic features of primary lung lesions were added compared to the model based solely on the clinical features, but without statistical significance (*P* = 0.425). The predictive model with primary lung lesions showed slightly higher AUC compared to the added consideration of metastatic lesions, but without statistical significance (P = 0.203).

### Survival Analyses

**Figure 3:**
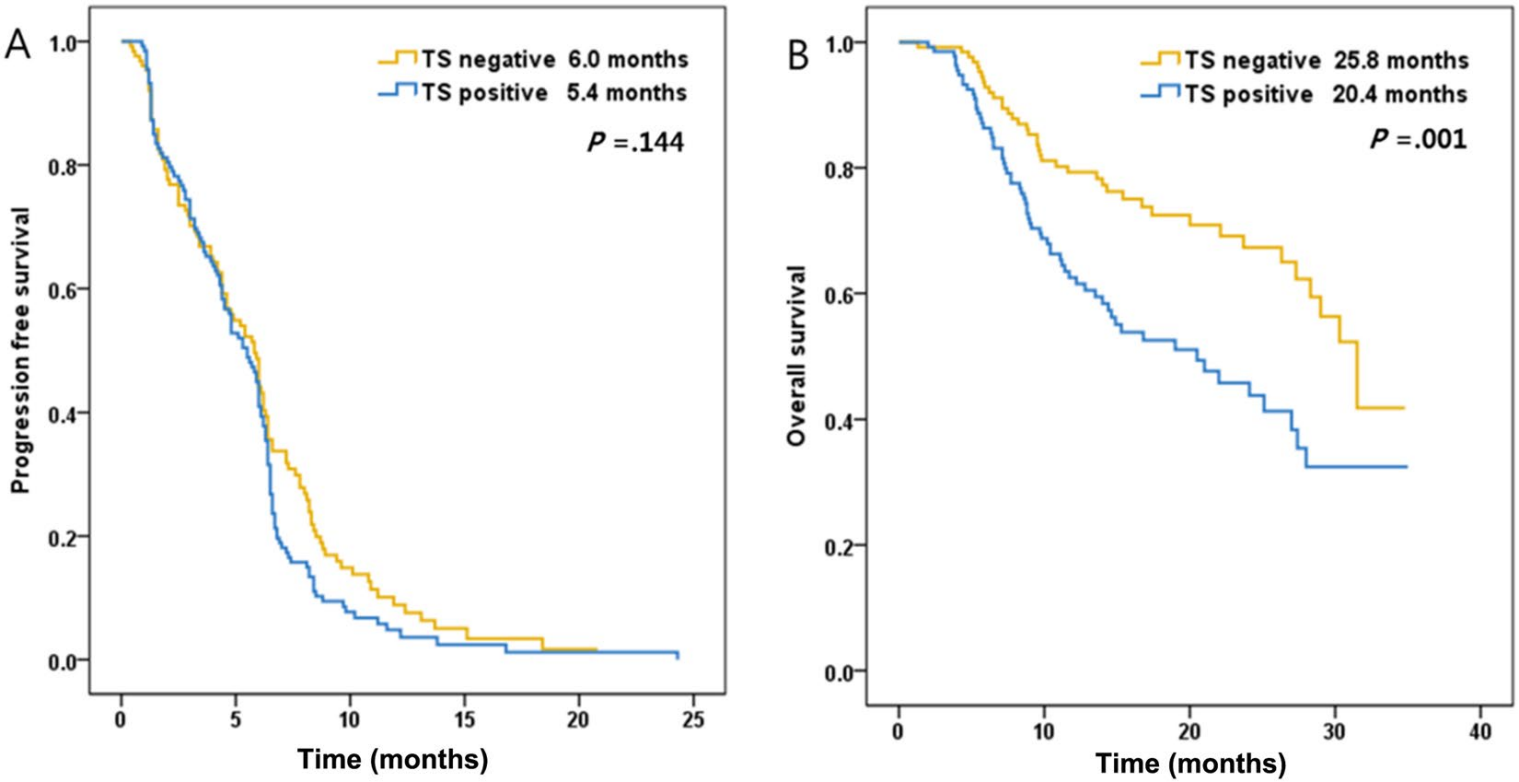
Progression-free survival (PFS, A) and overall survival (OS, B) curves according to TS expression status. The PFS was not significantly associated with TS expression status (*P* = 0.144). However, the OS was significantly prolonged in the TS-negative group compared with the TS-positive group (*P* = 0.001).

The correlations between TS expression status and PFS and OS in 260 patients with non-squamous NSCLC are shown in Fig. 3. The PFS was not significantly associated with TS expression status (*P* = 0.144). However, the OS was significantly prolonged in the TS-negative group compared with the TS-positive group (*P* = 0.001).

## Discussion

Recent studies have reported that low TS expression level is associated with better clinical outcome and longer overall survival^8–10,13–15,17^. Consistent with these previous reports, our study also suggests that TS-negativity is a good prognostic factor. A few studies have shown controversial results that high TS expression level was associated with improved survival^31,32^. These discrepancies are mainly due to tumor heterogeneity. In other words, the result from a small specimen may not be indicative of the TS status of the entire tumor. Additionally, the invasive process of obtaining a specimen from the tumor and the non-standardized procedures to evaluate TS expression level remain critical points. The development of antibodies to detect TS in a sensitive and quantitative assay made it possible to measure the TS expression level in tumor samples using IHC^33^. However, TS evaluation with IHC is a semi-quantitative and empirical method that depends on the detailed procedures and is therefore difficult to standardize.

Considering these limitations in detecting TS expression level from tumor samples, predicting TS expression level based on clinicoradiologic factors determined in non-invasive studies such as CT or PET/CT images of the NSCLC patients is of great clinical importance. A previous study has described the clinicopathologic characteristics of TS-negative lung cancer, demonstrating that female, younger age, never-smoker status, and adenocarcinoma were more frequent in TS-negative patients^10^. Since these clinicopathologic characteristics are not specific for TS expression status, adding radiologic features may increase the predictive probability. Radiomic features from CT or PET are widely being explored by recent studies for their correlation with prognosis, treatment response or tumor phenotype including EGFR-mutation status in NSCLC patients^34–36^. Our study also presents that some CT radiomic features were univariately predictive of TS expression status. However, the addition of radiomic features to clinical factors did not show significant improvement in predicting TS-negativity. CT radiomic features have potential for predicting TS expression status, but there is no clinical significance beyond the data variability of clinicoradiologic features. SUVmax from PET/CT was not significantly correlated with TS expression status. To our knowledge, there has been no previous description of the radiologic characteristics of lung cancers with TS stratification, and our study is the first attempt to better understand both clinical and radiologic characteristics of TS-positive lung adenocarcinomas.

We determined that radiomic features including skewness, kurtosis (histogram-based features), entropy, and dissimilarity (GLCM-based features) from primary lung lesions reflecting tumoral heterogeneity were significantly correlated with TS expression level. Histogram-based features reflect intensity information of a given ROI and quantify intra-tumor heterogeneity. Lower skewness (a measure of asymmetry of a histogram) and higher kurtosis (a measure of flatness of a histogram) representing increased heterogeneity were observed in both the total and outer ROI values of TS-positive tumors. In NSCLC, intra-tumor heterogeneity reflected by CT texture analysis is known to be associated with MAPK tumor pathway and has potential as a prognostic indicator^37^. Win *et al*. suggested the intra-tumor genomic heterogeneity or tumor hypoxia as the probable reasons for the relationship between tumoral heterogeneity and prognosis^38^. In previous studies, both kurtosis and skewness were associated with v-Ki-ras2 Kirsten rat sarcoma viral oncogene homolog (*KRAS*) mutation, and epidermal growth factor receptor (*EGFR*) mutation status was also correlated with CT texture features^39,40^. Tumor heterogeneity reflects the distribution of CT pixel values of the tumor and is related to tumor aggression. Additionally, there is emerging evidence that tumor segmentation of NSCLC on CT may improve prediction of survival (e.g., necrosis in the core and proliferation along the boundary)^21^. In our study, we partitioned the outer 1/3 of the ROI, and the mean, median, skewness, and kurtosis of the outer ROI and the maximum of the delta ROI were significant predictors of TS expression status. We made an attempt to enhance the value of the tumor partitioning for more detailed evaluation of tumor texture analysis. For validation of these findings, tumor partitioning must be applied in a larger study.

Whereas histogram-based features reflect intra-tumor heterogeneity in terms of intensity, GLCM-based features, which are textural features, reflect the heterogeneity of spatial distribution of voxel intensities. These features consider intensity values of a neighborhood instead of a single voxel. In our study, entropy and dissimilarity (GLCM-based features) of primary lung lesions were significantly correlated with TS expression level. Entropy indicates the uncertainty of the GLCM, which reflects the randomness of the matrix. Dissimilarity indicates how different each element is in the matrix.

Recently, with the development of targeted therapies in NSCLC, determining tumor mutation status is an important part of diagnosis in many countries to improve treatment outcomes^41^. However, in advanced lung cancer patients, therapeutic decision-making based on the results from primary tumor specimens could be justified only in cases where the tumor mutation status is similar between primary and metastatic lesions^42^. On the contrary, it is not always possible to obtain primary tumor tissue due to the invasiveness of the procedure, and a metastatic lesion may be obtained instead of the primary tumor^43^. Nevertheless, there is controversy over the concordance between the primary and metastatic lesions of NSCLC with multiple mutation statuses, including *EGFR*, *KRAS*, *p16*, and *p53*^44–51^. Generally, substantial mutation status concordance was observed between primary and metastatic lesions in previous studies, with the concordance varying from 60 to 100%^51^. Potential causes contributing to some level of mutation status discordance include the testing methodology, site of the metastatic sample, and tumor heterogeneity^51^. Tumor heterogeneity exists not only within a single tumor (intratumor), but also between tumors of the same type in a patient (intertumor)^42^. Although there is a close genetic relationship between the primary and metastatic lesions, cancer is an evolving systemic disease, and the primary and metastatic lesions could become divergent as they evolve. In a previous study by Shimizu *et al*.^52^, patients with EGFR mutations in both primary and metastatic tumors showed a higher disease control rate compared to the patients with EGFR mutations in primary tumors only (P = 0.062). This suggests that heterogeneity of mutation status between the primary and metastatic tumors may influence the treatment efficacy.

Allowing for these current concepts regarding primary and metastatic tumors, whether or not metastatic lesions should be included in the analysis of radiomic features is an important issue. At least, in NSCLC, there has been no attempt to investigate TS expression in metastases and compare it with the matched primary tumor. Several studies have been published on the correlation between TS expression in the primary tumor and metastatic lesions in colorectal cancer patients^53–59^, where the majority of the studies revealed a poor correlation of TS expression between the primary tumor and distant metastases^53–56,59^. Discordance of TS expression level in the primary tumor tissue with the outcome of FU-based chemotherapy for metastatic disease suggests that TS analysis must be performed on biopsy material obtained from the metastatic site or sites^60^. Here, our study demonstrated that clinicoradiologic characteristics of primary lung lesion did not significantly better reflect the TS expression status obtained from the primary lung lesion itself in comparison with the combined data of primary lung and metastatic lesions. However, the potential variability of TS expression between different tissues raises concern over the use of primary tumor tissue to predict treatment response or prognosis in metastatic disease. Further and preferably prospective studies are needed to evaluate TS expression in NSCLC metastatic lesions in comparison with matched primary lung lesions and to correlate findings with responsiveness or prognosis.

A potential limitation of our study is its retrospective design from a single institution. However, the patients originally participated in a phase II trial and were prospectively stratified into TS-positive or -negative groups. CT or PET/CT exams performed on a several different multi-detector CT scanners and various organs included for radiologic characteristics evaluation in metastases might be other potential limitations. Regardless of heterogeneity in CT scanners or protocols, we minimized the effect of CT acquisition to radiomic feature analysis by including CT images with thin slice thicknesses (2.5 mm or less) and standard reconstruction algorithm. Further studies with a larger sample size of patients from multiple institutions are required to validate our results.

In conclusion, TS-negativity is a potential prognostic biomarker, and our study presents that although CT radiomic features have potential for predicting TS expression status, clinical significance in uncertain. The addition of radiomic features to clinical factors did not show significant improvement in predicting TS-negativity.

## Electronic supplementary material

Supplementary Appendix

Mat code

Mat code

Mat code

Mat code
