# Supplementary material for: Deciphering Clinicoradiologic Phenotype for Thymidylate Synthase Expression Status in Patients with Advanced Lung Adenocarcinoma Using a Radiomics Approach
Source: Sci Rep. 2018 Jun 12;8:8968. doi: 10.1038/s41598-018-27273-9 (PMC5997691; doi:10.1038/s41598-018-27273-9)
Supplement: Supplementary file 1 — Supplementary Appendix [file 41598_2018_27273_MOESM1_ESM.docx]

**Deciphering Clinicoradiologic Phenotype for Thymidylate Synthase Expression Status in Patients with Advanced Lung Adenocarcinoma Using a Radiomics Approach**

So Won Lee, MD^1,2,*^, Hyunjin Park, PhD^3,4,*^, Ho Yun Lee, MD^1,+^, Insuk Sohn, PhD^5^, Seung-Hak Lee MS^6^, Jun Kang, MD^7^, Jong-Mu Sun, MD^8^, Myung-Ju Ahn, MD^8^

^1^Department of Radiology and Center for Imaging Science, Samsung Medical Center, Sungkyunkwan University School of Medicine, Seoul, Korea

^2^Department of Radiology, CHA Gangnam Medical Center, CHA University, Seoul, Korea

^3^School of Electronic and Electrical Engineering, Sungkyunkwan University, Suwon, Korea

^4^Center for Neuroscience Imaging Research, Institute for Basic Science, Suwon, Korea

^5^Statistics and Data Center, Samsung Medical Center, Seoul, Korea

^6^Department of Electronic Electrical and Computer Engineering, Sungkyunkwan University, Suwon, Korea

^7^Department of Pathology, Inchun St. Mary's Hospital, College of Medicine, Catholic University of Korea, Inchun, Korea

^8^Division of Hematology-Oncology, Department of Internal Medicine, Samsung Medical Center, Sungkyunkwan University School of Medicine, Seoul, Korea

**Supplementary Appendix 1**

PET/CT imaging was performed using dedicated PET/CT scanners (Discovery LS or Discovery STe; GE Healthcare). Patients fasted for at least 6 h before the scan, and blood glucose levels, which were measured before the injection of 18F-FDG, were required to be less than 200 mg/dL. Unenhanced whole-body CT was performed with a continuous spiral technique with 8-slice helical CT (140 keV; 40–120 mA; section width, 5 mm) at 60 min after injection of 18F-FDG for Discovery LS scanner and with 16-slice helical CT (140 keV; 30–170 mA; section width, 3.75 mm) for Discovery STe scanner. Attenuation-corrected PET images were reconstructed using CT for attenuation correction with an ordered-subset expectation maximization algorithm (4.3 × 4.3 × 3.9 mm, 28 subsets, 2 iterations for Discovery LS scanner; 3.9 × 3.9 × 3.3 mm, 20 subsets, 2 iterations for Discovery STe scanner).

**Supplementary Figure 1**


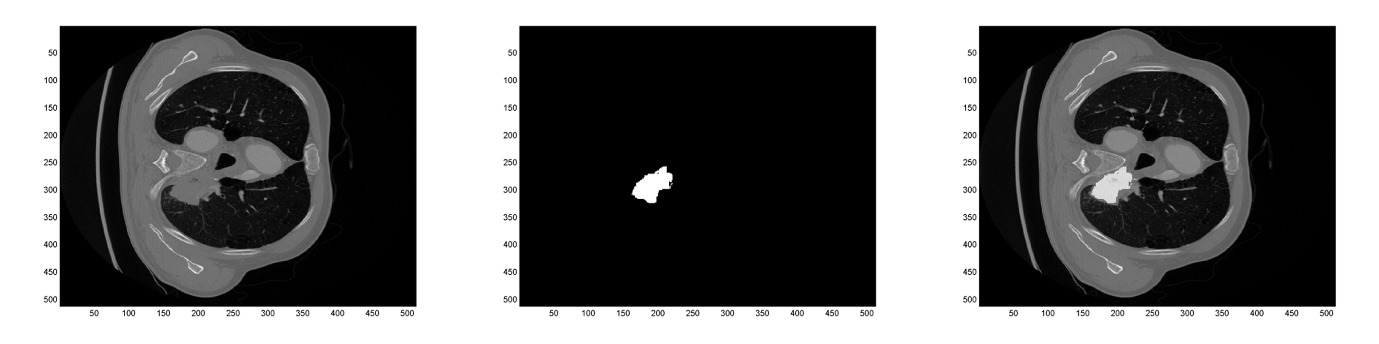


Fig S1. Sample ROI contouring. Left: Original CT image; Middle: ROI image; Right: CT image + ROI image (Combined). ROIs are 3D but here only a representative 2D slice is shown.

**Supplementary Figure 2**


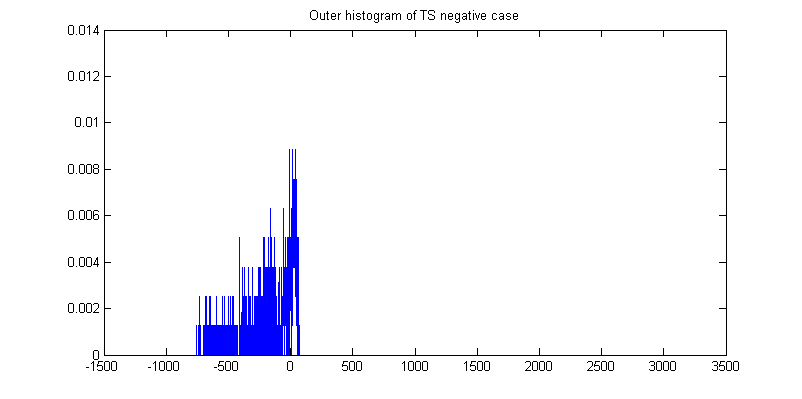


Sample histogram of outer ROI for TS-, whole tumor: Kurtosis value = 3.93, Skewness value = -1.36 Outer tumor: Kurtosis value =2.92, Skewness = -0.93


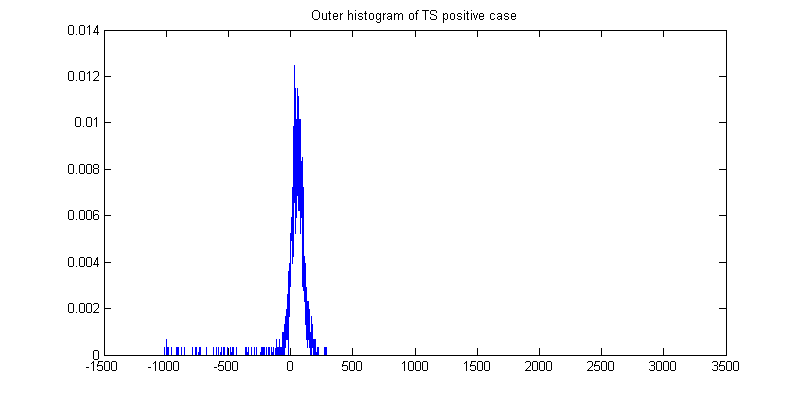


Sample histogram of outer ROI for TS+, Whole tumor: Kurtosis value = 87.10, Skewness value = -7.16 Outer tumor: Kurtosis value = 59.78, Skewness = -6.38

**Supplementary Table 1**

Reproducibility of ROI contouring and feature extraction was estimated by comparing the intraclass correlation coefficient (ICC) values between the two radiologists in 49 randomly selected patients (supplementary table) . The range of ICC values was 0.657 to 0.999, with mean a value of 0.877, representing a moderate or higher reliability level of agreement.

| **Variables** | **ICC** | **95% CI** | |
| --- | --- | --- | --- |
| **mean_val** | 0.921 | 0.813 | 0.962 |
| **std_val** | 0.799 | 0.643 | 0.886 |
| **Volume_val** | 0.999 | 0.998 | 0.999 |
| **Density_val** | 0.921 | 0.813 | 0.962 |
| **Mass_val** | 0.999 | 0.998 | 0.999 |
| **var_val** | 0.792 | 0.631 | 0.883 |
| **Max_val** | 0.802 | 0.651 | 0.888 |
| **Median_val** | 0.948 | 0.873 | 0.975 |
| **Min_val** | 0.751 | 0.561 | 0.859 |
| **IQR_val** | 0.864 | 0.761 | 0.923 |
| **Range_val** | 0.813 | 0.669 | 0.895 |
| **RMS_val** | 0.891 | 0.802 | 0.939 |
| **Skewness** | 0.944 | 0.897 | 0.969 |
| **Entropy_val4096** | 0.910 | 0.841 | 0.949 |
| **Kurtosis_val** | 0.885 | 0.797 | 0.935 |
| **Uniformity_val4096** | 0.940 | 0.894 | 0.966 |
| **UPP_val4096** | 0.859 | 0.597 | 0.937 |
| **percentile_histogram 2.5%** | 0.813 | 0.669 | 0.894 |
| **percentile_histogram 25%** | 0.899 | 0.803 | 0.946 |
| **percentile_histogram 50%** | 0.948 | 0.873 | 0.975 |
| **percentile_histogram 75%** | 0.947 | 0.812 | 0.978 |
| **percentile_histogram 97.5%** | 0.761 | 0.367 | 0.891 |
| **mean_val_out** | 0.884 | 0.755 | 0.940 |
| **std_val_out** | 0.687 | 0.444 | 0.823 |
| **var_val_out** | 0.657 | 0.392 | 0.807 |
| **Max_val_out** | 0.735 | 0.508 | 0.855 |
| **Median_val_out** | 0.886 | 0.766 | 0.940 |
| **Min_val_out** | 0.716 | 0.498 | 0.840 |
| **Skewness_val_out** | 0.918 | 0.849 | 0.955 |
| **Entropy_val4096_out** | 0.924 | 0.866 | 0.957 |
| **Kurtosis_val_out** | 0.797 | 0.642 | 0.885 |
| **mean_val_in** | 0.933 | 0.750 | 0.973 |
| **std_val_in** | 0.818 | 0.675 | 0.898 |
| **var_val_in** | 0.783 | 0.609 | 0.878 |
| **Max_val_in** | 0.879 | 0.786 | 0.932 |
| **Median_val_in** | 0.959 | 0.825 | 0.984 |
| **Min_val_in** | 0.780 | 0.602 | 0.878 |
| **Skewness_val_in** | 0.801 | 0.647 | 0.888 |
| **Entropy_val4096_in** | 0.904 | 0.829 | 0.946 |
| **Kurtosis_val_in** | 0.725 | 0.511 | 0.846 |
| **convexity** | 0.935 | 0.679 | 0.976 |
| **Surface_area** | 0.991 | 0.978 | 0.996 |
| **Compactness_val1** | 0.991 | 0.983 | 0.995 |
| **Max3d_dia_val** | 0.972 | 0.942 | 0.985 |
| **Spherical_disproportion_val** | 0.900 | 0.783 | 0.949 |
| **Sphericity_val** | 0.901 | 0.738 | 0.954 |
| **SVR** | 0.952 | 0.913 | 0.973 |
| **Auto_corr_val** | 0.900 | 0.823 | 0.944 |
| **Cluster_tendency_val** | 0.900 | 0.823 | 0.944 |
| **Contrast_glcm_val** | 0.936 | 0.886 | 0.964 |
| **Diff_entropy_val** | 0.925 | 0.866 | 0.958 |
| **Dissimilarity_val** | 0.930 | 0.875 | 0.961 |
| **Entropy_glcm_val** | 0.867 | 0.765 | 0.925 |
| **Energy_glcm_val** | 0.854 | 0.742 | 0.918 |
| **Homogeneity_val1** | 0.923 | 0.864 | 0.956 |
| **IMC1_val** | 0.984 | 0.970 | 0.991 |
| **Max_probability_val** | 0.882 | 0.791 | 0.933 |
| **Variance_glcm_val** | 0.900 | 0.822 | 0.943 |
| **size_zone_var_32** | 0.958 | 0.916 | 0.977 |
| **inten_var_32** | 0.850 | 0.730 | 0.917 |

**Supplementary Table 2**

A total of 60 CT radiomic features used in this study

| **Classes of texture features** | **Based methods** | **Parameter** | **Formula** | **Description** | |
| --- | --- | --- | --- | --- | --- |
| **Statistical based** | **1^st^ order features** (Histogram based features) | Maximum | $Max=max(X\left( i \right))$  Where $X$ denote the 3d image matrix | Measures maximum intensity value of a histogram | |
|  |  | Minimum | $Min=min(X\left( i \right))$  Where $X$ denote the 3d image matrix | Measures minimum intensity value of a histogram | |
|  |  | Median | $Median=\frac{X(i)}{2}$  Where $X$ denote the 3d image matrix | Measures median intensity value of a histogram | |
|  |  | Mean | $Mean=\frac{1}{N}\sum_{i}^{N} X(i)$  Where $X$ denote the 3d image matrix with $N$ voxel. | Measures mean intensity value of a histogram | |
|  |  | Variance | $Variance=\frac{1}{N-1}\sum_{i=1}^{N} \left( X\left( i \right)-\bar{x} \right)^{2}$ | Measures squared distances of each value of a histogram from the mean | |
|  |  | Standard deviation | $Std=\left( \frac{1}{N-1}\sum_{i=1}^{N} \left( X\left( i \right)-\bar{x} \right)^{2} \right)^{1/2}$  Where $X$ denote the 3d image matrix with $N$ voxel. | Measures amount of variation of a histogram. | |
|  |  | Skewness | $Skewness=\frac{E{(x-\mu)}^{3}}{\sigma^{3}}$  Where $\mu$ is the mean of $x$, $\sigma$ is the standard deviation of $x$, $E$ is the expectation operator. | Measures asymmetry of a histogram. | |
|  |  | Kurtosis | $Kurtosis=\frac{{E(x-\mu)}^{4}}{\sigma^{4}}$  Where $\mu$ is the mean of$x$, $\sigma$ is the standard deviation of $x$, $E$ is the expectation operator. | Measures “peakedeness” of a histogram (flatness of histogram) | |
|  |  | Root mean square (RMS) | $RMS=\sqrt{\frac{1}{N}\sum_{n=1}^{N} \left\vert X_{n} \right\vert^{2}}$  Where $X$ denote the 3d image matrix with $N$ voxel. | Measures the square-root of the mean of the squares of the values of the histogram. This feature is another measure of the magnitude of a histogram | |
|  |  | Inter quartile range | $IQR=Q_{3}-Q_{1}$  Where $Q_{3}$ denote the 3^rd^ quartile of histogram, $Q_{1}$ denote the 1^st^ quartile of histogram | Measures of variability, based on dividing a histogram into quartiles | |
|  |  | Range | $Range=range(X\left( i \right))$ | Measures difference between the highest and lowest voxel values of a histogram | |
|  |  | Entropy | $Entropy=-\sum_{i=1}^{N_{l}} P(i)\log_{2} P(i)$  Where $P$ denote the first order histogram with $N_{l}$ discrete intensity levels. | Measures irregularity of a histogram. | |
|  |  | Uniformity | $Uniformity=\sum_{i=1}^{N_{l}} {P(i)}^{2}$  Where $P$ denote the first order histogram with $N_{l}$ discrete intensity levels. | Measures uniformity of a histogram. | |
|  |  | Uniformity value of positive pixels (UPP) | $UPP=\sum_{i=1}^{N_{l}} \left\vert P(i) \right\vert^{2}$  Where $P$ denote the first order histogram with $N_{l}$ discrete intensity levels. | Measures uniformity of positive values of a histogram | |
|  |  | Percentile | $Percentile=\left( \frac{n^{th} percentile}{100} \right) X(i)$ | Measures intensity value at the 2.5^th^ , 25^th^ ,50^th^ ,75^th^ , and 97.5^th^ percentile on histogram | |
|  | **Higher order features**  (GLCM based features) | Autocorrelation | $Autocorrelation =\sum_{i=1}^{N_{g}} \sum_{j=1}^{N_{g}} \mathrm{ij}\mathbf{P}(i,j)$ | Measures of the magnitude of the fineness and coarseness of texture | |
|  |  | Cluster tendency | $Cluster tendency=$  $\sum_{i=1}^{N_{g}} \sum_{j=1}^{N_{g}} \left[ i+j-\mu_{x}\left( i \right) -\mu_{y}\left( j \right) \right]^{2}\mathbf{P}(i,j)$ | Measures number of potential clusters present | |
|  |  | Maximum probability | $Maximum probability=max\{P\left( i,j \right)\}$ | Measures maximum value of GLCM matrix | |
|  |  | Contrast | $Contrast=\sum_{i=1}^{N_{g}} \sum_{j=1}^{N_{g}} \left\vert i-j \right\vert^{2}\mathbf{P}(i,j)$ | Measures of the local intensity variation of GLCM | |
|  |  | Difference entropy | $Difference entropy=\sum_{i=0}^{N_{g}-1} \mathbf{P}_{x-y}(i)\log_{2} [P_{x-y}(i)]$ | Measures entropy of processed GLCM matrix Px-y | |
|  |  | Dissimilarity | $Dissimilarity=\sum_{i=1}^{N_{g}} \sum_{j=1}^{N_{g}} \left\vert i-j \right\vert\mathbf{P}(i,j)$ | Measures differences of entries in GLCM | |
|  |  | Energy | $Energy=\sum_{i=1}^{N_{g}} \sum_{j=1}^{N_{g}} \left[ \mathbf{P}\left( i,j \right) \right]^{2}$ | Measures of the homogeneity of GLCM | |
|  |  | Entropy | $Entropy=-\sum_{i=1}^{N_{g}} \sum_{j=1}^{N_{g}} \mathbf{P}(i,j)\log_{2} [\mathbf{P}\left( i,j \right)]$ | Measures irregularity of GLCM | |
|  |  | Homogeneity | $Homogeneity=\sum_{i=1}^{N_{g}} \sum_{j=1}^{N_{g}} \frac{\mathbf{P}\left( i,j \right)}{1+\left\vert i-j \right\vert}$ | Measures closeness of GLCM | |
|  |  | Informational measure of correlation (IMC) | $IMC=HXY-\frac{HXY1}{max\{HX,HY\}}$ | Secondary measure of Homogeneity1 | |
|  |  | Variance | $Variance=\sum_{i=1}^{N_{g}} \sum_{j=1}^{N_{g}} \left( i-\mu\right)^{2}P(i,j)$ | Measures dispersion of the parameter values around the mean of the combinations of reference and neighborhood pixels | |
|  |  | Image intensities are discretized to 256 levels for robust computation of the GLCM. GLCMs were computed for 13 directions and the average of 13 matrices were used for feature computation.  Where $\mathbf{P}\left( i,j \right)$is the gray level co-occurrence matrix for ($\delta=1, \alpha=0)$,  $N_{g}$is the number of discrete intensity value in the image,  $\mu$ is the mean of $\mathbf{P}\left( i,j \right),$  $p_{x}\left( i \right)=\sum_{j=1}^{N_{g}} \mathbf{P}(i,j)$ is the marginal row probabilities,  $p_{y}\left( i \right)=\sum_{i=1}^{N_{g}} \mathbf{P}(i,j)$ is the marginal column probabilities,  $\mu_{x}$ is the mean of $p_{x}$,  $\mu_{y}$ is the mean of $p_{y}$,  $\sigma_{x}$ is the standard deviation of $p_{x}$,  $\sigma_{y}$ is the standard deviation of $p_{y}$,  $p_{x+y}\left( k \right)=\sum_{i=1}^{N_{g}} \sum_{j=1}^{N_{g}} \mathbf{P}\left( i,j \right) , i+j=k, k=2,3,\ldots,2N_{g}$,  $p_{x-y}\left( k \right)=\sum_{i=1}^{N_{g}} \sum_{j=1}^{N_{g}} \mathbf{P}\left( i,j \right) ,\left\vert i-j \right\vert=k, k=0,1,\ldots,N_{g}-1$,  $HX=-\sum_{i=1}^{N_{g}} \mathbf{P}_{x}(i)\log_{2} \left[ p_{x}(i) \right]$ is the entropy of $\mathbf{P}_{x}$,  $HY=-\sum_{i=1}^{N_{g}} \mathbf{P}_{y}(i)\log_{2} \left[ p_{y}(i) \right]$ is the entropy of $\mathbf{P}_{y}$,  $H=\sum_{i=1}^{N_{g}} \sum_{j=1}^{N_{g}} \mathbf{P}\left( i,j \right)\log_{2} \left[ \mathbf{P}(i,j) \right]$is the entropy of $\mathbf{P}\left( i,j \right)$  $HXY=-\sum_{i=1}^{N_{g}} \sum_{j=1}^{N_{g}} \mathbf{P}\left( i,j \right)\log(p_{x}\left( i \right)p_{y}\left( j \right))$,  $HXY=-\sum_{i=1}^{N_{g}} \sum_{j=1}^{N_{g}} p_{x}(i)p_{y}(j)\log(p_{x}\left( i \right)p_{y}\left( j \right))$. | | | |
|  | **Higher order features**  (ISZ based features) | Size-zone variability | $\frac{1}{\Theta}{\sum_{m=1}^{M} \left[ \sum_{n=1}^{N} \mathbf{P}\left( m,n \right) \right]}^{2}$ | | Variability in the size |
|  |  | Intensity variability | $\frac{1}{\Theta}{\sum_{n=1}^{N} \left[ \sum_{m=1}^{M} \mathbf{P}\left( m,n \right) \right]}^{2}$ | | Variability in the intensity |
|  |  | Image intensities are discretized to 32 levels for robust computation of the ISZ matrix.  Where $\boldsymbol{P}\left( m,n \right)$ is the intensity size zone matrix  $\Theta$ represents the number of homogeneous areas in tumor,  $M$ is the number of distinct intensity values,  $N$ is the size of homogeneous area in the matrix $\boldsymbol{P}\left( m,n \right)$ | | | |
| **Morphological features** | **Shape and Size based features** | Compactness | $Compactness=\frac{V}{\sqrt{\pi}A^{\frac{2}{3}}}$  Where$V$ denote the volume and $A$ denote the surface area of the volume of interest (VOI) | | Quantifies how close an object to the smoothest shape, the circle |
|  |  | Surface area | $SA=\sum_{i=1}^{N} \frac{1}{2}\left\vert a_{i}b_{i}\times a_{i}c_{i} \right\vert$  Where $N$ is the total number triangle (coved surface area) and $a,b, c$ are edge vectors | | The surface area of the ROI |
|  |  | Convexity | $Convexity=\frac{V}{V^{'}}$  Where $V$ denote tumor volume and $V^{'}$ denote convex hull volume | | Measures ratio of the ROI volume contained within the tumor to the calculated convex hull volume |
|  |  | Sphericity | $Sphericity=\frac{36\pi\times{{(V}^{2})}^{\frac{1}{3}}}{A}$  Where $A$ denote area and $V$ denote tumor volume | | Measures of the roundness of the ROI |
|  |  | Maximum 3D diameter | See description in the next column | | Measures of the maximum 3D ROI diameter. It is measured as the largest pairwise Euclidean distance, between surface voxels of the ROI |
|  |  | Spherical disproportion | $Spherical disproportion=\frac{A}{4\pi R^{2}}$  Where $R$ is the radius of a sphere with the same volume as the ROI | | The ratio of the surface area of the ROI to the surface area of a sphere with the same volume as the ROI |
|  |  | Surface to volume ratio (SVR) | $SVR=\frac{A}{V}$  Where $A$ is area and $V$ is volume | | Surface to volume ratio |
|  |  | Volume | $Volume=R*number of voxels$  Where $R$ denote the 3d image resolution | | Volume of tumor (ROI) |
|  |  | Mass | $Mass=V*D$  Where $V$ denote the tumor volume, $D$ denote the tumor density | | Mass of tumor (ROI) |
|  |  | Density | $Density=\frac{M}{V}$  Where $V$ denote the tumor volume, $M$ denote the tumor mass | | Density of tumor (ROI) |

**Supplementary Table 3**

Number of features in each category

| **Statistical based** | **1^st^ order features** | Whole ROI | Maximum, Minimum, Median, Mean, Variance, , Standard deviation, Skewness, Kurtosis, Root mean square, Inter quartile range, Range, Entropy, Uniformity, , Uniformity value of positive pixels, Percentile (2.5^th^ , 25^th^ ,50^th^ ,75^th^ , and 97.5^th^ percentile) | 19features |
| --- | --- | --- | --- | --- |
|  |  | ROI outer | Mean, Standard deviation, Variance, Maximum, Median, Minimum, Skewness, Kurtosis, Entropy, Energy | 9 features |
|  |  | ROI delta | Mean, Standard deviation, Variance, Maximum, Median, Minimum, Skewness, Kurtosis, Entropy, Energy | 9 features |
|  | **Higher order features**  (GLCM based features) | Whole ROI | Each set of ROI had 11 features (Autocorrelation, Cluster tendency, Maximum probability, Contrast, Difference entropy, Dissimilarity, Energy, Entropy, Homogeneity, Informational measure of correlation (IMC), Variance) | 11 features |
|  | **Higher order features**  (ISZ based features) | Whole ROI | Size-zone variability, Intensity variability | 2 features |
| **Morphological features** | **Shape and Size based features** | Whole ROI | Compactness, Surface area, Convexity, Sphericity, Maximum 3D diameter, Spherical disproportion, Surface to volume ratio (SVR), Volume, Mass, Density | 10 features |

**Supplementary Table 4**

Correlation between volume and CT derived texture features.

| **Features** | **Correlation (r)** | **P-value** |
| --- | --- | --- |
| **Auto_corr_val** | -0.2898 | 0.00013217 |
| **Cluster_tendency_val** | -0.2921 | 0.00011666 |
| **Contrast_glcm_val** | -0.3478 | 0.0000035908 |
| **Diff_entropy_val** | 0.3745 | 0.00000052892 |
| **Dissimilarity_val** | -0.3607 | 0.0000014507 |
| **Entropy_glcm_val** | -0.3277 | 0.000013681 |
| **Energy_glcm_val** | 0.1041 | 0.1780 |
| **Homogeneity_val1** | 0.2606 | 0.00062083 |
| **IMC1_val** | 0.4622 | 0.00000000025181 |
| **Max_probability_val** | 0.0762 | 0.3245 |
| **Variance_glcm_val** | -0.2951 | 0.000098099 |

**Supplementary Data**

The source codes used for the analysis.

**Main code**

%% Code for extracting the radiomics features, not in pyradiomics

% This is a research code and the user needs to modify the code as necessary

% Our code was developed using MATLAB version R2017a

%% Reference

% [1] Intratumor heterogeneity characterized by textural features on baseline 18 F-FDG PET images predicts response to concomitant radiochemotherapy in esophageal cancer (The Journal of nuclear medicine)

% [2] Pulmonary Ground-Glass Nodules: Increase in Mass as an Early Indicator of Growth (Radiology)

% [3] Solitary Pulmonary Nodular Lung Adenocarcinoma: Correlation of Histopathologic Scoring and Patient Survival with Imaging Biomarkers (Radiology)

%% TS case code for required features

clear;clc; close all; whitebg('white');

% Patient number

pat_num = strcat(temp_pat_num)

fname_img = strcat(pat_num,'.img');

fname_roi = strcat('l',pat_num,'.img');

% Read in CT nii (NIFTI) file.

% This line requires NIFIT toolbox ver 1.27

t1 = load_nii(fname_img);

% Read in ROI file. ROI is a binary (0/1) nii file

roi = load_nii(fname_roi);

% Process the ROI. 0 is the background and 1 is the target

idx = find(roi.img > 0);

[idx_i, idx_j, idx_k] = ind2sub(size(roi.img), idx);

inten_list = double(t1.img(idx));

%% Histogram based features

% Uniformity of distribution of positive gray-level voxel values (UPP)

hist_bin_center = -1024:3071;

hist1 = hist(inten_list(:), hist_bin_center);

hist1 = hist1./sum(hist1);

hist1_sub = hist1(1026:end);

hist1_sub2 = hist1_sub./sum(hist1_sub);

UPP_val = sum(hist1_sub2.^2);

% Percentile

percentile_histogram = prctile(inten_list,[2.5 25 50 75 97.5]);

%% Intensity-size zone features (32bins) [1]. This requires a separate function rad_ISZ (provided separately)

feature_ISZ = rad_ISZ_ts(t1,roi,32); %isz_features = [size_zone_variability_ISZ, intensity_zone_variability_ISZ];

%% SHAPE features. This requires a separate function rad_SHAPE (provided separately)

% Convexity

feature_SHAPE_ts = rad_SHAPE_ts(roi);

% Volume

resol_x = t1.hdr.dime.pixdim(2); resol_y = t1.hdr.dime.pixdim(3); resol_z = t1.hdr.dime.pixdim(4);

D_resol=(resol_x*resol_y*resol_z);

Volume_val = length(inten_list)*D_resol;

% Density [2]

mean_val = mean(inten_list );

Density_val = Volume_val*(mean_val+1000);

% Mass [3]

Mass_val = Density_val*Volume_val;

%% Save all features (.xls, .mat)

save(strcat('radiomics_ts_',pat_num))

val=[percentile_histogram, UPP_val, feature_SHAPE_ts, Density_val, Mass_val, feature_ISZ];

xlswrite(strcat('pat_',pat_num,'_radiomics_ts.xlsx'), val, 1, 'A2');

**Shape function code**

function shape_features = rad_SHAPE_ts(roi_nii)

% Function : rad_SHAPE_ts

% Calculate convexity feature

% Input : roi_nii

% Output : 1 feature

% [convexity_SHAPE]

% Volume

unit_volume = roi_nii.hdr.dime.pixdim(2)*roi_nii.hdr.dime.pixdim(3)*roi_nii.hdr.dime.pixdim(4);

volume_SHAPE = unit_volume*length(find(roi_nii.img));

% Convexity

[roi_idx_i,roi_idx_j,roi_idx_k] = ind2sub(size(roi_nii.img),find(roi_nii.img));

[K,convex_volume] = convhull(roi_idx_i,roi_idx_j,roi_idx_k);

convex_volume = convex_volume*unit_volume;

convexity_SHAPE = volume_SHAPE/convex_volume;

%% feature output

shape_features = [convexity_SHAPE];

end**Intensity-size zone function code**

function isz_features = rad_ISZ_ts(image_nii,roi_nii,NUM_BIN_ISZ)

% Function : rad_ISZ_ts

% Calculate ISZM based features

% Input : image_nii,roi_nii,NUM_BIN_ISZ

% Output : 2 features

% [size_zone_variability_ISZ, intensity_zone_variability_ISZ]

ISZM = rad_ISZ_build_ISZM_6_ts(image_nii,roi_nii,NUM_BIN_ISZ);

% Size zone variability

size_zone_variability_ISZ = rad_ISZ_size_zone_variability(ISZM);

% Intensity zone variability

intensity_zone_variability_ISZ = rad_ISZ_intensity_zone_variability(ISZM);

isz_features = [size_zone_variability_ISZ, intensity_zone_variability_ISZ];

end

**Intensity-size zone matrix build code**

function ISZM = rad_ISZ_build_ISZM_6_ts(image_nii,roi_nii,NUM_BIN_ISZ)

%% Build Intensity Size Zone Matrix using 6 direction DFS

MAX_ZONE_SIZE = 256;

ISZM = zeros(NUM_BIN_ISZ,MAX_ZONE_SIZE);

%% Binning

roi_idx = find(roi_nii.img);

image_binned = nan(size(image_nii.img));

image_binned(roi_idx) = image_nii.img(roi_idx) - min(image_nii.img(roi_idx));

rng = range(image_binned(:));

image_binned = floor(image_binned/(rng/NUM_BIN_ISZ));

image_binned(find(image_binned == max(image_binned(:)))) = NUM_BIN_ISZ - 1;

image_binned = image_binned + 1;

%% Calculate ISZ

roi_visit_map = zeros(size(roi_nii.img));

length_roi_idx = length(roi_idx);

roi_idx_idx = 1;

tic

while roi_idx_idx <= length_roi_idx

if roi_visit_map(roi_idx(roi_idx_idx)) == 0

roi_visit_map(roi_idx(roi_idx_idx)) = 1;

[gray_level,zone_size,roi_visit_map] = rad_ISZ_DFS4ISZM_BUILD(roi_idx(roi_idx_idx),roi_visit_map,image_binned,roi_nii);

if zone_size > MAX_ZONE_SIZE

zone_size = MAX_ZONE_SIZE;

end

ISZM(gray_level,zone_size) = ISZM(gray_level,zone_size) + 1;

end

roi_idx_idx = roi_idx_idx + 1;

end

toc

end
